# Triglyceride–glucose index as a key predictor of ARDS in acute pancreatitis: SHAP analysis reveals its critical role in risk stratification

**DOI:** 10.3389/fnut.2025.1662379

**Published:** 2025-11-21

**Authors:** Ju Luo, Zhe Chen, Cuirong Guo, Yingjie Su, Ning Ding

**Affiliations:** 1 Department of Geriatrics, The Affiliated Changsha Central Hospital, Hengyang Medical School, University of South China, Changsha, Hunan, China; 2 Department of Emergency Medicine, The Affiliated Changsha Central Hospital, Hengyang Medical School, University of South China, Changsha, Hunan, China

**Keywords:** triglyceride-glucose index, ARDS, acute pancreatitis, machine learning, prognosis

## Abstract

**Background:**

The relationship between triglyceride–glucose (TyG) index and acute respiratory distress syndrome (ARDS) in acute pancreatitis (AP) patients is still lacking. This study aimed to explore the association between the TyG index and ARDS in AP patients using an 8-year retrospective dataset.

**Methods:**

This study was performed in Changsha Central Hospital from January 2015 to December 2022. Univariate analysis was done to discuss the relationship between different characteristics and ARDS in AP. Multivariate regression analysis was employed to investigate the relationship between the TyG index and ARDS in AP. Eight machine learning models were employed to assess the in-hospital ARDS risk in AP patients. The SHapley Additive exPlanations (SHAP) method was utilized to verify the importance of TyG in the models.

**Results:**

A total of 2,382 AP patients were finally enrolled, and ARDS occurred in 137 patients. With per-unit increment in TyG index, the risk of ARDS in AP increased by 133%(OR = 2.33, 95%CI: 1.51–3.60, *p* = 0.0001) after adjusting all potential confounders. The relationship between the TyG index and ARDS in AP was non-linear. The XGBoost (AUC = 0.857 ± 0.034) and Random Forest (AUC = 0.851 ± 0.045) algorithms were the best two performance methods. In the SHAP analysis, TyG was the second most important feature in the RF model and the seventh in the XGBoost model.

**Conclusion:**

TyG index was associated with in-hospital ARDS in AP. The XGBoost and Random Forest models based on the TyG index had the best performance for predicting ARDS in AP patients. The SHAP method further confirmed that the TyG index serves as a significant predictor for the development of ARDS in patients with acute pancreatitis.

## Introduction

Acute pancreatitis (AP) is a general digestive disease with a comparatively high morbidity, which is characterized by abdominal symptoms and increased pancreatic enzymes, partly leading to organ dysfunction and life-threatening condition (1). Due to the regional differences, the incidence of AP varies from 30 to 40 cases/100,000 persons every year globally (2, 3). Around 20% of the AP patients might develop into severe acute pancreatitis (SAP), which is characterized by persistent organ failure and a high mortality increase from 20 to 40% (4, 5), thereby increasing both the therapeutic challenges and economic burden of the disease.

Previous studies demonstrate that in SAP, the respiratory system is usually affected, and ARDS could be one of the most common organ dysfunction disorders (3, 6). About one-third of SAP patients develop ARDS, accounting for approximately 60% of deaths in the first week of onset (7, 8). Recently, although there have been many advances in the treatment of ARDS, its prognosis is still poor (9). Therefore, early identification of high-risk patients with early intervention and treatment to prevent and delay the occurrence and development of ARDS is significant for improving patient outcomes.

Previous researches show that some predictive models, including the Lung Injury Prediction Score (LIPS) and the Early Acute Lung Injury (EALI) score, have been used to evaluate the risk of ARDS from various disorders (3, 10). However, these scoring systems lack specificity in predicting the occurrence of ARDS with AP. However, most of the models developed in previous studies to predict ARDS related to SAP contain excessive parameters, relatively complex calculations, and strong dependence on imaging or unconventional clinical examinations, which greatly limit their clinical application (2, 11, 12).

The triglyceride glucose (TyG) index is derived from fasting triglyceride (TG) and blood glucose (FBG) levels and has become a simple alternative biomarker for insulin resistance (IR). An increasing amount of research evidence suggests that IR plays a critical role in AP and its complications, and the TyG index is associated with the prognosis of SAP (3, 13). Zhang et al. reported that in animal models of ARDS, it was found that insulin signaling was significantly downregulated, and IR played a crucial role in the occurrence and development of ARDS (14). Another study included 206 patients with COVID-19, and the results suggest that the TyG index is closely related to the severity and prognosis of ARDS (15). However, there is still a lack of research on the relationship between the TyG index and ARDS in AP patients.

The aim of this study is to explore the association between the TyG index and ARDS in AP patients using data collected over 8 years, providing a new biomarker for identifying early ARDS in AP patients and promoting timely clinical intervention.

## Methods

### Study cohort

**Figure 1:**
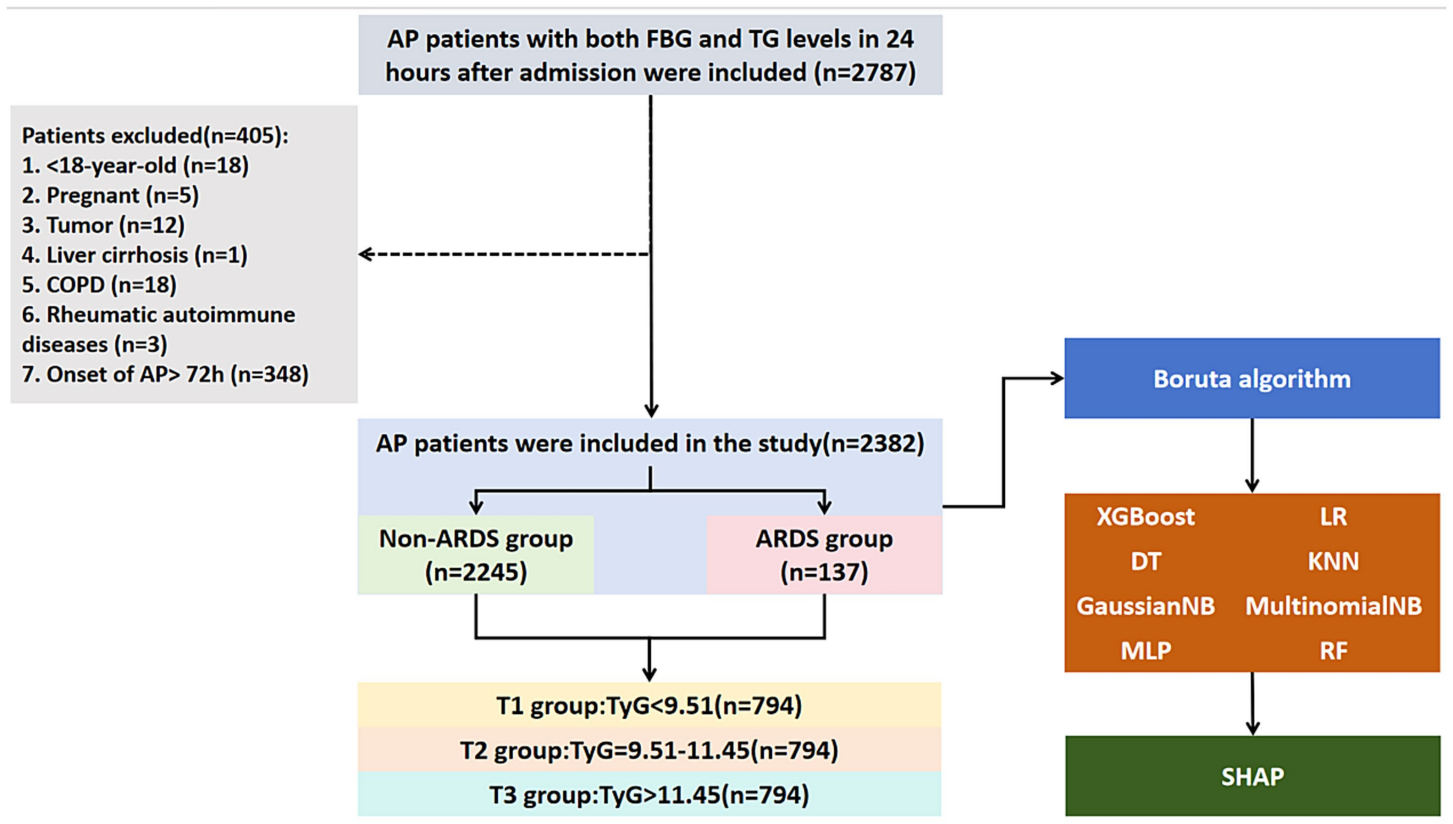
Flow chart for patients’ enrollment and study design. ARDS, acute respiratory distress syndrome; AP, acute pancreatitis; TG, triglyceride; FBG, fasting blood glucose; COPD, chronic obstructive pulmonary disease; XGBoost, eXtreme Gradient Boosting; LR, Logistic Regression; DT, Decision Tree; KNN, K-Nearest Neighbors; GaussianNB, Gaussian Naive Bayes; MultinomialNB, Multinomial Naive Bayes; MLP, Multilayer perceptron; RF, Random Forest; SHAP, SHapley Additive exPlanations.

This retrospective study was performed on the basis of the clinical data from Changsha Central Hospital. In the study, all the AP patients with records of fasting blood sugar (FBG) and triglyceride (TG) within 24 h after admission to the hospital from January 2015 to December 2022 were enrolled (*n* = 2,787) (Figure 1). Patients with following criteria were excluded (*n* = 405): (1) < 18-year-old (*n* = 18); (2) pregnant (*n* = 5); (3) tumor (*n* = 12); (4) liver cirrhosis (*n* = 1); (5) chronic obstructive pulmonary disease (COPD) (*n* = 18); (6) rheumatic autoimmune diseases (*n* = 3); (7) onset of AP > 72 h (*n* = 348) (Figure 1). Finally, 2,383 AP patients were included.

### Definitions

The definition of AP was confirmed when at least two out of three these criteria were met as follows: (1) digestive symptoms such as abdominal pain or abdominal distention; (2) the levels of serum amylase or lipase increased by three times compared with the normal levels of serum amylase or lipase; (3) ultrasonic or CT/MRI scan findings show the AP (16, 17). The etiology of hypertriglyceridemia (HTG) in AP was confirmed as follows: (1) TG ≥ 1,000 mg/dL; (2) TG ≥ 500 mg/dL with milky serum (18, 19). The definition of ARDS was based on the Berlin definition (20) and the new global definition of ARDS (2021) (21).

### Exposure and endpoint

TyG index was defined by {ln [TG(mg/dL) × FBG(mg/dL)/2]}. The endpoint was in-hospital ARDS.

### Characteristics

Baseline characteristics, such as age, gender, etiology (HTG, biliary), comorbidity [diabetes, hypertension, and coronary artery disease (CAD)], vital signs [heart rate (HR), systolic blood pressure (SBP), respiratory rate (RR), diastolic blood pressure (DBP)], and length of stay (LOS) in hospital were included. Lab findings within 24 h after admission in hospital were collected: TG, FBG, white blood cell (WBC), creatinine, aspartate aminotransferase (AST), platelet (PLT), total bilirubin, prothrombin time (PT), albumin, globulin, alanine aminotransferase (ALT), urea nitrogen, amylase, lipase, total cholesterol, total calcium, high-density lipoprotein (HDL), ionized calcium, and low-density lipoprotein (LDL). Scores within 24 h after admission to the hospital were collected: Ranson, bedside index of severity in acute pancreatitis (BISAP), acute physiology, and chronic health evaluation (APACHEII).

### Statistical analysis

We used the Packages R^1^ and EmpowerStats^2^ software for statistical analysis. Statistically significant difference was considered with the *p*-value < 0.05.

First, on the basis of the tertiles of the TyG index, the study cohort was divided into three groups (T1–T3 groups). Baseline characteristics between T1 and T3 groups were analyzed. Continuous characteristics were expressed by the median with interquartile ranges (Median, Q1–Q3). Categorical characteristics were shown by the percentages (Number, %). Mann–Whitney U-test or Chi-squared test was used. Second, univariate analysis was done to discuss the relationship between different characteristics and ARDS in AP. Third, multivariate regression analysis was utilized to investigate the relationship between the TyG index and ARDS in AP. Three different models were performed as follows: Crude model (adjusted for none), Model I (adjusted for gender, age, diabetes, hypertension, CAD, HTG, biliary), Model II (adjusted for gender, age, comorbidities, etiology, vital signs, laboratory variables, scores of Ranson, BISAP, and APACHEII). Fourth, we explored whether the relationship between TyG and ARDS in AP was a linear or non-linear feature. The non-linear model was considered with the *p*-value less than 0.05. Moreover, the generalized additive model was used for performing the smooth fitting curve. Fifth, the receiver-operator characteristic (ROC) with accuracy, sensitivity, and specificity of the TyG index was used for predicting ARDS in AP. In addition, subgroup analysis was performed.

### Machine learning method

For dealing with missing data, the multiple imputation method Miceforest was utilized (22).

It is based on the multiple imputation of the chain equation of random forest using the process of predictive mean matching to select the value to be estimated. The Boruta algorithm in machine learning was used to rank the features according to their importance of the predictive ability of in-hospital ARDS. We use the random forest chain equation for multiple imputation and employ a predictive mean matching process to select estimated values. We use the Boruta algorithm in machine learning to assess the importance of predictive ability for ARDS in hospitalized patients and to rank features accordingly.

Then, the acceptable variables are integrated into the machine learning (ML) algorithm. The Logistic Regression (LR), eXtreme Gradient Boosting (XGBoost), K-Nearest Neighbors (KNN), Decision Tree (DT), Multinomial Naive Bayes (MultinomialNB), Random Forest (RF), Multilayer perceptron (MLP), and Gaussian Naive Bayes (GaussianNB) were used to evaluate the in-hospital ARDS risk of AP patients. The patient dataset was randomly divided into a development set and a validation set in an 8:2 ratio. A fivefold cross validation is used for internal verification. ROC curve and AUC are used to evaluate the performance of the model. The SHapley Additive exPlans (SHAP) method is used to validate the role of TyG in the model. All relevant machine learning methods and codes can be accessed for free at the following website https://github.com/philiplaw1984/TyG/.

## Results

### Baseline characteristics of the AP patient’s cohort

Based on the flow chart (Figure 1), 405 patients were excluded and 2,382 AP patients were finally enrolled in this study. In the study cohort, the median age was 43 years, and men accounted for 74.06% (*n* = 1764) (Table 1). The number of AP patients based on the different etiologies, such as alcohol, HTG, and biliary were 509 (21.37%), 1,234 (51.81%), and 423 (17.76%), respectively. The number of patients with diabetes, hypertension, and CAD was 458 (19.23%), 436 (18.30%), and 72 (3.02%), respectively. The median TyG index was 10.58 (9.14–11.81).

**Table 1:** Comparison of general variables between ARDS group and non-ARDS group in AP patients.

Variables	Total	Non-ARDS group	ARDS group	*P*-value
Number	2,382	2,245	137	
Age (years) (median, IQR)	43.00 (35.00–54.00)	43.00 (35.00–55.00)	40.00 (34.00–49.00)	0.095
Etiology (*n*,%)				
HTG	1,234 (51.81%)	1,136 (50.60%)	98 (71.53%)	<0.001
Biliary	423 (17.76%)	411 (18.31%)	12 (8.76%)	<0.001
Gender (*n*,%)				0.007
Female	618 (25.94%)	596 (26.55%)	22 (16.06%)	
Male	1764 (74.06%)	1,649 (73.45%)	115 (83.94%)	
Comorbidity (*n*,%)				
Diabetes	458 (19.23%)	421 (18.75%)	37 (27.01%)	0.017
Hypertension	436 (18.30%)	408 (18.17%)	28 (20.44%)	0.056
CAD	72 (3.02%)	67 (2.98%)	5 (3.65%)	0.659
Characteristics (median, IQR)				
SBP (mmHg)	136.00 (123.00–152.00)	135.00 (123.00–152.00)	138.00 (124.00–151.50)	0.705
DBP (mmHg)	82.00 (74.00–90.00)	81.00 (74.00–90.00)	85.00 (76.75–93.00)	0.025
HR (beats/min)	84.00 (74.50–96.00)	83.00 (74.00–95.00)	100.50 (85.00–114.75)	<0.001
RR (beats/min)	20.00 (20.00–20.00)	20.00 (20.00–20.00)	20.00 (20.00–22.00)	<0.001
TyG index	10.58 (9.14–11.81)	10.50 (9.10–11.73)	12.05 (10.26–12.68)	<0.001
TG (mg/dL)	563.30 (132.75–1685.04)	518.61 (128.32–1609.82)	1831.95 (307.10–2473.57)	<0.001
FBG (mg/dL)	142.20 (115.20–196.20)	140.40 (113.40–192.60)	178.20 (142.20–291.60)	<0.001
WBC (*10^9^/L)	12.14 (9.06–15.19)	11.96 (8.97–14.92)	15.37 (12.08–19.20)	<0.001
PLT (*10^9^/L)	218.00 (178.00–264.00)	218.00 (178.00–264.00)	215.00 (179.50–252.50)	0.597
PT (s)	11.40 (10.70–12.00)	11.40 (10.70–12.00)	11.70 (10.80–12.50)	<0.001
Albumin (g/L)	44.00 (41.00–47.00)	44.00 (41.00–47.00)	44.00 (39.95–46.68)	0.156
Globulin (g/L)	30.00 (26.00–35.00)	30.00 (26.00–35.00)	32.00 (28.00–36.62)	0.003
ALT (IU/L)	33.00 (20.00–60.00)	33.00 (20.00–60.00)	32.50 (24.00–60.00)	0.634
AST (IU/L)	27.00 (20.00–49.00)	27.00 (20.00–49.00)	31.00 (23.75–48.00)	0.674
Total bilirubin (umol/L)	12.60 (8.30–20.00)	12.50 (8.20–19.80)	14.95 (10.00–22.55)	0.326
Urea nitrogen (mmol/L)	4.41 (3.55–5.49)	4.40 (3.55–5.48)	4.51 (3.60–5.92)	<0.001
Creatinine (umol/L)	67.00 (56.00–78.00)	67.00 (55.40–78.00)	69.00 (60.00–80.25)	<0.001
Amylase (IU/L)	199.00 (83.00–680.00)	193.00 (81.00–627.75)	369.00 (112.00–1088.00)	0.007
Lipase (IU/L)	311.00 (100.00–863.00)	296.00 (95.75–805.25)	607.00 (185.00–1643.00)	<0.001
Total calcium (mmol/L)	2.37 (2.26–2.47)	2.37 (2.27–2.47)	2.34 (2.15–2.48)	<0.001
Ionized calcium (mmol/L)	1.23 (1.18–1.28)	1.23 (1.18–1.28)	1.22 (1.12–1.29)	<0.001
Total cholesterol (mmol/L)	6.17 (4.77–9.14)	6.08 (4.76–8.80)	9.55 (5.35–12.99)	<0.001
HDL (mmol/L)	0.87 (0.60–1.25)	0.88 (0.60–1.26)	0.65 (0.44–0.91)	0.007
LDL (mmol/L)	2.47 (1.63–3.30)	2.50 (1.66–3.30)	2.19 (1.40–3.31)	0.311
LOS in hospital (days)	6.00 (4.00–8.00)	6.00 (4.00–8.00)	10.00 (7.00–15.00)	<0.001
Scoring systems (IQR)				
Ranson	1.00 (1.00–2.00)	1.00 (1.00–2.00)	2.00 (1.00–3.00)	<0.001
BISAP	1.00 (0.00–1.00)	1.00 (0.00–1.00)	1.00 (1.00–2.00)	<0.001
APACHEII	7.00 (5.00–10.00)	7.00 (5.00–10.00)	10.00 (6.00–14.00)	<0.001

ARDS, acute respiratory distress syndrome; AP, acute pancreatitis; TyG index, triglyceride glucose index; HTG, hypertriglyceridemia; CAD, coronary artery disease; SBP, systolic blood pressure; DBP, diastolic blood pressure; HR, heart rate; RR, respiratory rate; TG, triglyceride; FBG, fasting blood glucose; WBC, white blood cells; PLT, platelet; PT, prothrombin time; ALT, alanine aminotransferase; AST, aspartate aminotransferase; HDL, high density lipoprotein; LDL, low density lipoprotein; LOS, length of stay; APACHE, acute physiology and chronic health evaluation; BISAP, bedside index of severity in acute pancreatitis; IQR, interquartile ranges. Symbol * denotes the multiplication sign.

### Comparison of general variables between ARDS group and non-ARDS group in AP patients

In Table 1, general variables between the ARDS group (*n* = 137) and the non-ARDS group (*n* = 2,245) were compared. In the ARDS group, the ratio of HTG-AP was 71.58% (*n* = 98), and men accounted for 83.94% (*n* = 115) (both *p* < 0.05). The proportion of diabetes was significantly higher in the ARDS group (*p* = 0.017). Variables including DBP, HR, RR, TyG, TG, FBG, WBC, PT, globulin, urea nitrogen, creatinine, amylase, lipase, and total cholesterol were significantly higher in the ARDS group (all *p* < 0.05), while the levels of total calcium, ionized calcium, and HDL were significantly lower in the ARDS group (all *p* < 0.05). The LOS in hospital was longer and the scores of Ranson, BISAP, and APACHEII were higher in the ARDS group (all *p* < 0.001).

### Comparison of baseline characteristics between three groups based on tertiles of TyG index in AP patients

In Table 2, on the basis of the tertile levels of the TyG index, the AP patients were divided into three groups: T1 group (*n* = 794, TyG index<9.51), T2 group (*n* = 794, TyG index: 9.51–11.45), and T3 group (*n* = 794, TyG index>11.45). The incidences of ARDS in T1, T2, and T3 groups were 2.90% (*n* = 23), 3.40% (*n* = 27), and 10.96% (*n* = 87), respectively (*p* < 0.001). In the T3 group, the age was significantly lower, and the proportions of HTG, men, and diabetes were significantly higher (all *p* < 0.001). The median levels of TG, FBG, and TyG index in the T3 group were 2223.12 mg/dL, 198.00 mg/dL, and 12.15, respectively. The vital sign indexes, such as SBP, DBP, HR, and RR were all higher in the T3 group (all *p* < 0.001). Lab findings, such as WBC, PLT, PT, albumin, globulin, ALT, AST, total bilirubin, urea nitrogen, creatinine, amylase, lipase, total calcium, ionized calcium, total cholesterol, HDL, and LDL, were significantly different between the three groups.

**Table 2:** Comparison of baseline characteristics between three groups based on tertiles of TyG index in AP patients.

TyG index (tertiles)				
Variables	T1 (<9.51)	T2 (9.51–11.45)	T3 (>11.45)	*P*-value
Number	794	794	794	
Age (years) (median, IQR)	54.00 (40.00–65.00)	41.00 (34.00–52.00)	39.00 (33.00–46.00)	<0.001
Etiology (*n*,%)				
HTG	0 (0.00%)	474 (59.70%)	760 (95.72%)	<0.001
Biliary	278 (35.01%)	111 (13.98%)	34 (4.28%)	<0.001
Gender (*n*,%)				<0.001
Female	309 (38.92%)	163 (20.53%)	146 (18.39%)	
Male	485 (61.08%)	631 (79.47%)	648 (81.61%)	
Comorbidities (*n*,%)				
Diabetes	56 (7.05%)	107 (13.48%)	295 (37.15%)	<0.001
Hypertension	186 (23.43%)	147 (18.51%)	103 (12.97%)	<0.001
CAD	47 (5.92%)	19 (2.39%)	6 (0.76%)	<0.001
Characteristics (median, IQR)				
SBP (mmHg)	132.00 (120.00–149.00)	136.00 (125.00–152.00)	137.50 (125.00–157.00)	<0.001
DBP (mmHg)	80.00 (70.00–88.00)	83.00 (75.00–91.00)	84.00 (76.00–91.00)	<0.001
HR (beats/min)	80.00 (72.00–90.00)	82.00 (74.00–94.00)	88.00 (78.00–104.00)	<0.001
RR (beats/min)	20.00 (20.00–20.00)	20.00 (20.00–20.00)	20.00 (20.00–22.00)	<0.001
TyG index	8.78 (8.43–9.14)	10.58 (10.05–11.06)	12.15 (11.81–12.56)	<0.001
TG (mg/dL)	99.12 (73.45–133.63)	563.30 (314.62–920.18)	2223.12 (1662.91–2505.88)	<0.001
FBG (mg/dL)	126.00 (108.00–154.58)	133.20 (113.40–174.60)	198.00 (141.66–273.15)	<0.001
WBC (*10^9^/L)	10.80 (8.28–14.16)	12.19 (9.51–15.17)	13.00 (9.90–15.73)	<0.001
PLT (*10^9^/L)	217.00 (178.00–258.50)	213.00 (175.00–264.00)	224.00 (183.00–269.00)	<0.001
PT (s)	11.50 (10.90–12.20)	11.20 (10.60–11.90)	11.40 (10.60–12.00)	<0.001
Albumin (g/L)	42.00 (39.00–45.00)	44.00 (41.00–47.00)	45.45 (43.00–48.00)	<0.001
Globulin (g/L)	27.00 (24.00–31.00)	29.00 (26.00–33.00)	35.00 (30.00–40.00)	<0.001
ALT (IU/L)	38.00 (19.00–151.50)	33.00 (22.45–55.00)	30.00 (20.00–45.00)	<0.001
AST (IU/L)	38.00 (22.00–151.20)	26.00 (20.00–41.00)	25.00 (19.00–33.00)	<0.001
Total bilirubin (μmol/L)	16.00 (9.00–28.05)	12.40 (8.60–19.00)	11.00 (7.70–16.00)	<0.001
Urea nitrogen (mmol/L)	4.78 (3.67–6.00)	4.46 (3.70–5.51)	4.16 (3.36–5.00)	<0.001
Creatinine (μmmol/L)	66.00 (55.00–78.00)	70.00 (59.00–80.85)	65.00 (54.00–75.00)	0.002
Amylase (IU/L)	478.00 (106.00–1418.00)	180.50 (79.00–502.00)	140.70 (72.00–327.75)	<0.001
Lipase (IU/L)	577.50 (113.75–1403.00)	290.00 (95.00–814.00)	227.50 (96.75–517.00)	<0.001
Total calcium (mmol/L)	2.34 (2.23–2.42)	2.36 (2.28–2.47)	2.41 (2.29–2.52)	<0.001
Ionized calcium (mmol/L)	1.22 (1.16–1.26)	1.23 (1.19–1.28)	1.25 (1.19–1.31)	<0.001
Total cholesterol (mmol/L)	4.72 (4.00–5.49)	5.77 (4.89–7.00)	10.38 (8.19–13.00)	<0.001
HDL (mmol/L)	1.35 (1.08–1.70)	0.88 (0.69–1.10)	0.56 (0.44–0.70)	<0.001
LDL (mmol/L)	2.71 (2.19–3.41)	2.28 (1.50–3.05)	2.21 (1.37–3.41)	<0.001
LOS in hospital (days)	6.00 (5.00–9.00)	5.00 (4.00–7.00)	6.00 (4.00–8.00)	<0.001
Scoring systems (IQR)				
Ranson	1.00 (1.00–2.00)	1.00 (1.00–2.00)	1.00 (1.00–2.00)	0.002
BISAP	1.00 (0.00–1.00)	1.00 (0.00–1.00)	1.00 (0.00–1.00)	0.169
APACHEII	8.00 (6.00–11.00)	7.00 (5.00–10.00)	7.00 (5.00–10.00)	<0.001
Outcomes (*n*,%)				
ARDS	23 (2.90%)	27 (3.40%)	87 (10.96%)	<0.001

ARDS, acute respiratory distress syndrome; AP, acute pancreatitis; TyG index, triglyceride glucose index; HTG, hypertriglyceridemia; CAD, coronary artery disease; SBP, systolic blood pressure; DBP, diastolic blood pressure; HR, heart rate; RR, respiratory rate; TG, triglyceride; FBG, fasting blood glucose; WBC, white blood cells; PLT, platelet; PT, prothrombin time; ALT, alanine aminotransferase; AST, aspartate aminotransferase; HDL, high density lipoprotein; LDL, low density lipoprotein; LOS, length of stay; APACHE, acute physiology and chronic health evaluation; BISAP, bedside index of severity in acute pancreatitis; IQR, interquartile ranges.

### Univariate analysis for ARDS in AP patients

In Table 3, variables including men (*p* = 0.0074), HTG (*p* < 0.0001), biliary (*p* = 0.0058), diabetes (*p* = 0.0182), DBP (*p* = 0.0248), HR (*p* < 0.0001), RR (*p* < 0.0001), TyG index (*p* < 0.0001), TG (*p* < 0.0001), FBG (*p* < 0.0001), WBC (*p* < 0.0001), PT (*p* < 0.0001), globulin (*p* = 0.0045), urea nitrogen (*p* = 0.0061), creatinine (*p* < 0.0001), amylase (*p* = 0.0083), lipase (*p* = 0.0007), total calcium (*p* = 0.0001), ionized calcium (*p* = 0.0004), total cholesterol (*p* < 0.0001), HDL (*p* = 0.0003), Ranson (*p* < 0.0001), BISAP (*p* < 0.0001), and APACHEII (*p* < 0.0001) were associated with ARDS in AP patients.

**Table 3:** Univariate analysis for ARDS in AP patients.

Variables	Univariate (OR, 95%CI, *P*-value)
Age (years)	0.99 (0.98, 1.00), 0.0952
Gender	
Female	Ref.
Male	1.89 (1.19, 3.01), 0.0074
HTG	
No	Ref.
Yes	2.45 (1.68, 3.59), <0.0001
Biliary	
No	Ref.
Yes	0.43 (0.23, 0.78), 0.0058
Diabetes	
No	Ref.
Yes	1.60 (1.08, 2.37), 0.0182
Hypertension	
No	Ref.
Yes	1.16 (0.75, 1.78), 0.5062
CAD	
No	Ref.
Yes	1.23 (0.49, 3.11), 0.6594
SBP (mmHg)	1.00 (0.99, 1.01), 0.7047
DBP (mmHg)	1.01 (1.00, 1.03), 0.0248
HR (beats/min)	1.05 (1.03, 1.06), <0.0001
RR (beats/min)	1.41 (1.31, 1.52), <0.0001
TyG index	1.66 (1.45, 1.90), <0.0001
TG (mg/dL)	1.00 (1.00, 1.00), <0.0001
FBG (mg/dL)	1.01 (1.01, 1.01), <0.0001
WBC (*10^9^/L)	1.15 (1.11, 1.19), <0.0001
PLT (*10^9^/L)	1.00 (1.00, 1.00), 0.5967
PT (s)	1.22 (1.11, 1.35), <0.0001
Albumin (g/L)	0.98 (0.94, 1.01), 0.1548
Globulin (g/L)	1.02 (1.00, 1.03), 0.0045
ALT (IU/L)	1.00 (1.00, 1.00), 0.6034
AST (IU/L)	1.00 (1.00, 1.00), 0.6741
Total bilirubin (μmol/L)	1.00 (1.00, 1.01), 0.3292
Urea nitrogen (mmol/L)	1.06 (1.02, 1.10), 0.0061
Creatinine (μmmol/L)	1.01 (1.01, 1.01), <0.0001
Amylase (IU/L)	1.00 (1.00, 1.00), 0.0083
Lipase (IU/L)	1.00 (1.00, 1.00), 0.0007
Total calcium (mmol/L)	0.19 (0.08, 0.43), 0.0001
Ionized calcium (mmol/L)	0.05 (0.01, 0.25), 0.0004
Total cholesterol (mmol/L)	1.12 (1.08, 1.16), <0.0001
HDL (mmol/L)	0.49 (0.33, 0.72), 0.0003
LDL (mmol/L)	0.95 (0.85, 1.05), 0.3097
Ranson	1.42 (1.25, 1.62), <0.0001
BISAP	2.15 (1.78, 2.60), <0.0001
APACHEII	1.17 (1.13, 1.22), <0.0001

ARDS, acute respiratory distress syndrome; AP, acute pancreatitis; TyG index, triglyceride glucose index; HTG, hypertriglyceridemia; CAD, coronary artery disease; SBP, systolic blood pressure; DBP, diastolic blood pressure; HR, heart rate; RR, respiratory rate; TG, triglyceride; FBG, fasting blood glucose; WBC, white blood cells; PLT, platelet; PT, prothrombin time; ALT, alanine aminotransferase; AST, aspartate aminotransferase; HDL, high density lipoprotein; LDL, low density lipoprotein; APACHE, acute physiology and chronic health evaluation; BISAP, bedside index of severity in acute pancreatitis; OR, odds ratio; CI, confidential interval.

### Associations between the TyG index and ARDS in AP patients in different models

In Table 4, with a per-unit increment in the TyG index, the risk of ARDS in AP in the crude model, adjusted model I, and model II increased by 66% (OR = 1.66, 95%CI: 1.45–1.90, *p* < 0.0001), 166% (OR = 2.66, 95%CI: 2.03–3.50, *p* < 0.0001), and 133% (OR = 2.33, 95%CI:1.51–3.60, *p* < 0.0001), respectively. Compared to the T1 group, the ORs of ARDS in the T3 group in the crude model, adjusted model I, and model II were 4.13 (95%CI: 2.58–6.60, *p* < 0.0001), 7.30 (95%CI:2.88–18.49, *p* < 0.0001), and 5.64 (95%CI:1.50–21.21, *p* = 0.0104), respectively.

**Table 4:** Associations between TyG index and ARDS in AP patients in different models.

Exposure	Crude model OR (95%CI), *P*-value	Adjusted model I OR (95%CI), *P*-value	Adjusted model II OR (95%CI), *P*-value
ARDS			
TyG index	1.66 (1.45, 1.90), <0.0001	2.66 (2.03, 3.50), <0.0001	2.33 (1.51, 3.60), 0.0001
TyG index (tertiles)			
T1 (<9.51)	Ref.	Ref.	Ref.
T2 (9.51–11.45)	1.18 (0.67, 2.08), 0.5658	1.48 (0.76, 2.88), 0.2433	1.75 (0.71, 4.30), 0.2223
T3 (>11.45)	4.13 (2.58, 6.60), <0.0001	7.30 (2.88, 18.49), <0.0001	5.64 (1.50, 21.21), 0.0104
*P* for trend	<0.0001	<0.0001	0.0086

Crude model adjusted for: None; Model I adjusted for: gender, age, diabetes, hypertension, CAD, HTG, biliary. Model II adjusted for: gender, age, diabetes, hypertension, CAD, HTG, biliary, SBP, DBP, HR, RR, WBC, PLT, PT, albumin, globulin, ALT, AST, total bilirubin, urea nitrogen, creatinine, amylase, lipase, total calcium, ionized calcium, total cholesterol, HDL, LDL, Ranson, BISAP, APACHEII. ARDS, acute respiratory distress syndrome; AP, acute pancreatitis; TyG index, triglyceride glucose index; HTG, hypertriglyceridemia; CAD, coronary artery disease; SBP, systolic blood pressure; DBP, diastolic blood pressure; HR, heart rate; RR, respiratory rate; TG, triglyceride; FBG, fasting blood glucose; WBC, white blood cells; PLT, platelet; PT, prothrombin time; ALT, alanine aminotransferase; AST, aspartate aminotransferase; HDL, high density lipoprotein; LDL, low density lipoprotein; APACHE, acute physiology and chronic health evaluation; BISAP, bedside index of severity in acute pancreatitis; OR, odds ratio; CI, confidential interval.

### The linear and non-linear models for the relationship between TyG index and ARDS in AP patients

**Figure 2:**
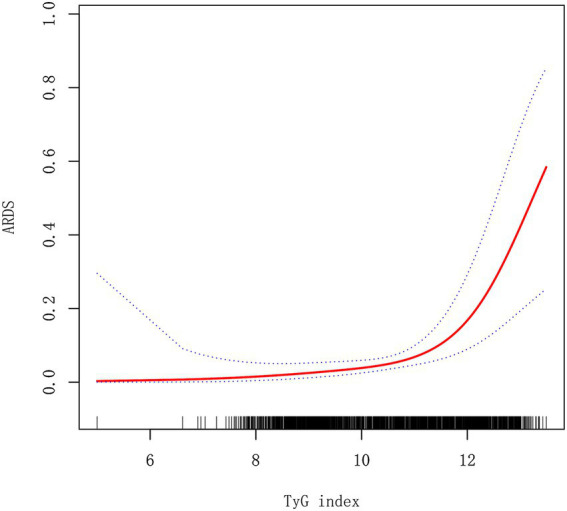
A smooth fitting curve shows the relationship between TyG index and ARDS in AP patients. ARDS, acute respiratory distress syndrome.

In Table 5, we compared the linear model and non-linear model, and the results showed that the non-linear relationship for indicating the association of TyG index and ARDS in AP was better [P for the log-likelihood ratio test was less than 0.05 (*p* = 0.010)]. When the TyG index≤11.31 (slope I), the OR was 1.40 (95%CI:0.79–2.48, *p* = 0.2528) (Figure 2). When the TyG index >11.31 (slope II), the OR was 4.30 (95%CI:2.26–8.19, *p* < 0.0001) (Figure 2). We also compared the two groups: TyG ≤ 11.31 (slope I) and TyG > 11.31 (slope II). The results showed that OR = 3.08 (95%CI: 1.31–7.25, *p* = 0.0100) when comparing slope II to slope I.

**Table 5:** The linear and non-linear models for the relationship between TyG index and ARDS in AP patients.

Models	Number (%)	OR (95%CI), *P*-value
The linear model	2,382 (100%)	2.33 (1.51, 3.60), 0.0001
The non-linear model		
The turning point of TyG index		11.31
≤11.31 (slope I)	1,514 (63.56%)	1.40 (0.79, 2.48), 0.2528
>11.31 (slope II)	868 (36.44%)	4.30 (2.26, 8.19), <0.0001
Slope II to slope I		3.08 (1.31, 7.25), 0.0100
Predicted at 11.31		−3.23 (−3.63, −2.82)
*P* for the log-likelihood ratio test		0.010

Both two models (linear model and non-linear model) adjusted for: gender, age, diabetes, hypertension, CAD, HTG, biliary, SBP, DBP, HR, RR, WBC, PLT, PT, albumin, globulin, ALT, AST, total bilirubin, urea nitrogen, creatinine, amylase, lipase, total calcium, ionized calcium, total cholesterol, HDL, LDL, Ranson, BISAP, APACHEII. ARDS, acute respiratory distress syndrome; AP, acute pancreatitis; TyG index, triglyceride glucose index; HTG, hypertriglyceridemia; CAD, coronary artery disease; SBP, systolic blood pressure; DBP, diastolic blood pressure; HR, heart rate; RR, respiratory rate; TG, triglyceride; FBG, fasting blood glucose; WBC, white blood cells; PLT, platelet; PT, prothrombin time; ALT, alanine aminotransferase; AST, aspartate aminotransferase; HDL, high density lipoprotein; LDL, low density lipoprotein; APACHE, acute physiology and chronic health evaluation; BISAP, bedside index of severity in acute pancreatitis; OR, odds ratio; CI, confidential interval.

### Subgroup analysis

In Table 6, subgroup analysis is shown. In AP with men (OR = 1.87, 95%CI: 1.58–2.21, *p* < 0.0001), low age (OR = 3.37, 95%CI: 2.25–5.06, *p* < 0.0001) and HTG (OR = 3.37, 95%CI: 2.41–4.70, *p* < 0.0001) had a higher risk of ARDS.

**Table 6:** Subgroup analysis.

Variables	OR (95%CI), *P*-value	*P* for interaction
Gender		0.0013
Female	1.10 (0.83, 1.46), 0.5209	
Male	1.87 (1.58, 2.21), <0.0001	
Age		<0.0001
Low	3.37 (2.25, 5.06), <0.0001	
Middle	1.58 (1.24, 2.01), 0.0002	
High	1.22 (0.95, 1.57), 0.1120	
CAD		0.3677
No	1.71 (1.48, 1.97), <0.0001	
Yes	1.25 (0.62, 2.49), 0.5325	
Diabetes		0.7008
No	1.72 (1.46, 2.02), <0.0001	
Yes	1.59 (1.14, 2.24), 0.0070	
Hypertension		0.1040
No	1.80 (1.53, 2.12), <0.0001	
Yes	1.39 (1.07, 1.81), 0.0151	
HTG		0.0011
No	1.42 (0.94, 2.13), 0.0937	
Yes	3.37 (2.41, 4.70), <0.0001	
Biliary		0.0807
No	1.72 (1.47, 2.01), <0.0001	
Yes	1.12 (0.69, 1.82), 0.6409	

HTG, hypertriglyceridemia; CAD, coronary artery disease; OR, odds ratio; CI, confidential interval.

### Importance of factors in the impact on in-hospital ARDS ranked by Boruta algorithm

**Figure 3:**
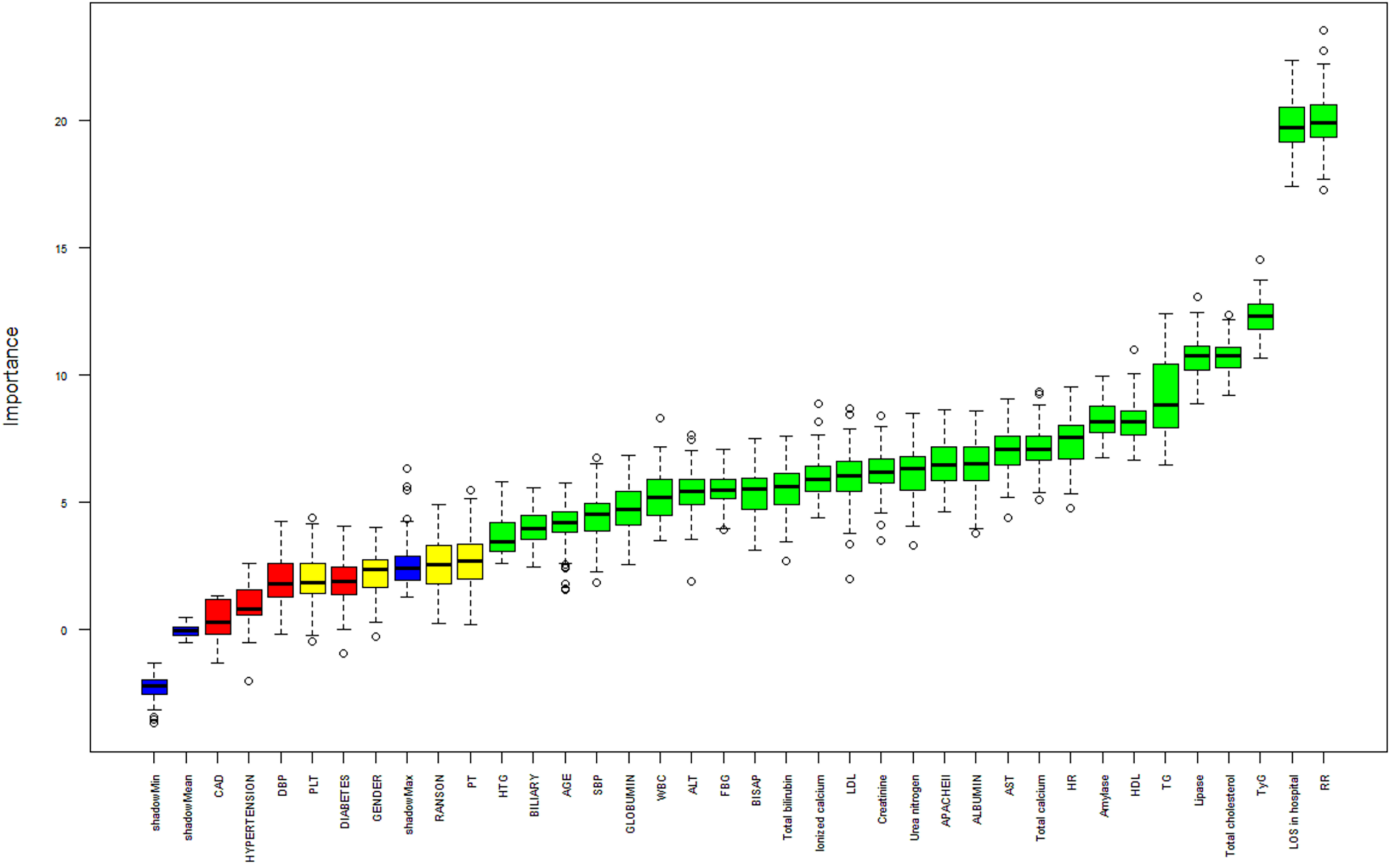
Importance of factors in the impact on in-hospital ARDS ranked by Boruta algorithm. ARDS, acute respiratory distress syndrome; AP, acute pancreatitis; TyG index, triglyceride glucose index; HTG, hypertriglyceridemia; CAD, coronary artery disease; SBP, systolic blood pressure; DBP, diastolic blood pressure; HR, heart rate; RR, respiratory rate; TG, triglyceride; FBG, fasting blood glucose; WBC, white blood cells; PLT, platelet; PT, prothrombin time; ALT, alanine aminotransferase; AST, aspartate aminotransferase; HDL, high density lipoprotein; LDL, low density lipoprotein; LOS, length of stay; APACHE, acute physiology and chronic health evaluation; BISAP, bedside index of severity in acute pancreatitis.

In the report from Boruta algorithm, RR, LOS in hospital, TyG, total cholesterol, lipase, TG, and all the variables in the green area were defined as important factors, which demonstrate the important roles in the model. On the contrary, the variables in the yellow and red areas were tentative and rejected factors. As shown in Figure 3, TyG was one of the key factors in the impact on in-hospital ARDS, ranked by Boruta algorithm.

### Establishment and validation of the ML models

**Figure 4:**
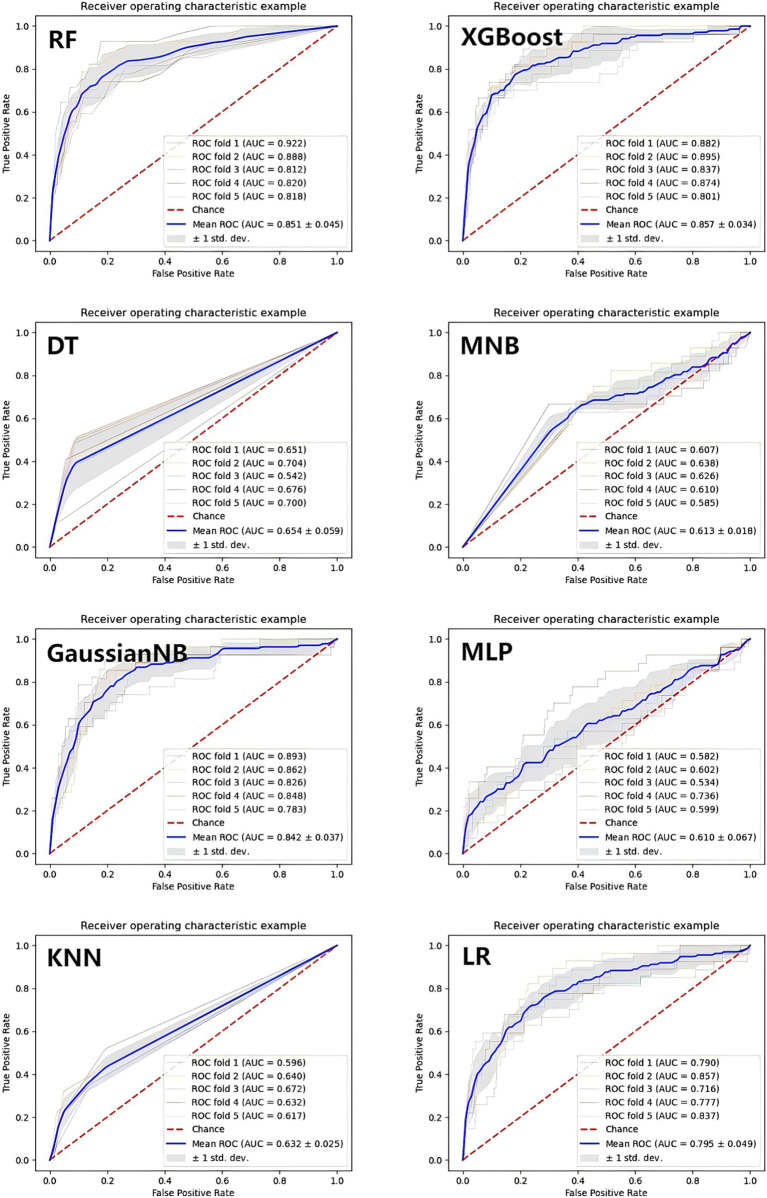
The ROC curves of the machine learning prediction model. XGBoost, eXtreme Gradient Boosting; LR, Logistic Regression; DT, Decision Tree; KNN, K-Nearest Neighbors; GaussianNB, Gaussian Naive Bayes; MultinomialNB, Multinomial Naive Bayes; MLP, Multilayer perceptron; RF, Random Forest.

In Figure 4, the ROC curves of different ML prediction models are illustrated. The AUCs of RF, XGBoost, DT, MNB, GaussianNB, MLP, KNN, and LR were 0.851 ± 0.045, 0.857 ± 0.034, 0.654 ± 0.059, 0.613 ± 0.018, 0.842 ± 0.037, 0.610 ± 0.067, 0.632 ± 0.025, and 0.795 ± 0.049, respectively, which indicated that the Random Forest and XGBoost models were the best two performance models. To further evaluate the model’s performance in excluding the two components (TG and FBG) that constitute the TyG index. We retrained the XGBoost and Random Forest models, and the AUC values of these two models were 0.850 ± 0.036 and 0.843 ± 0.056, respectively. The results were shown in Supplementary Figure S1. In order to strengthen the generalizability and credibility, we conducted further experiments and generated the Precision-Recall curve, Decision curve, and Calibration curve based on the XGBoost model. The results are shown in Supplementary Figure S2. A comparative analysis revealed that the XGBoost model outperformed the BISAP scoring system in predicting ARDS with significant improvement. The results are shown in Supplementary Figure S3.

### Interpretability for ML models

**Figure 5:**
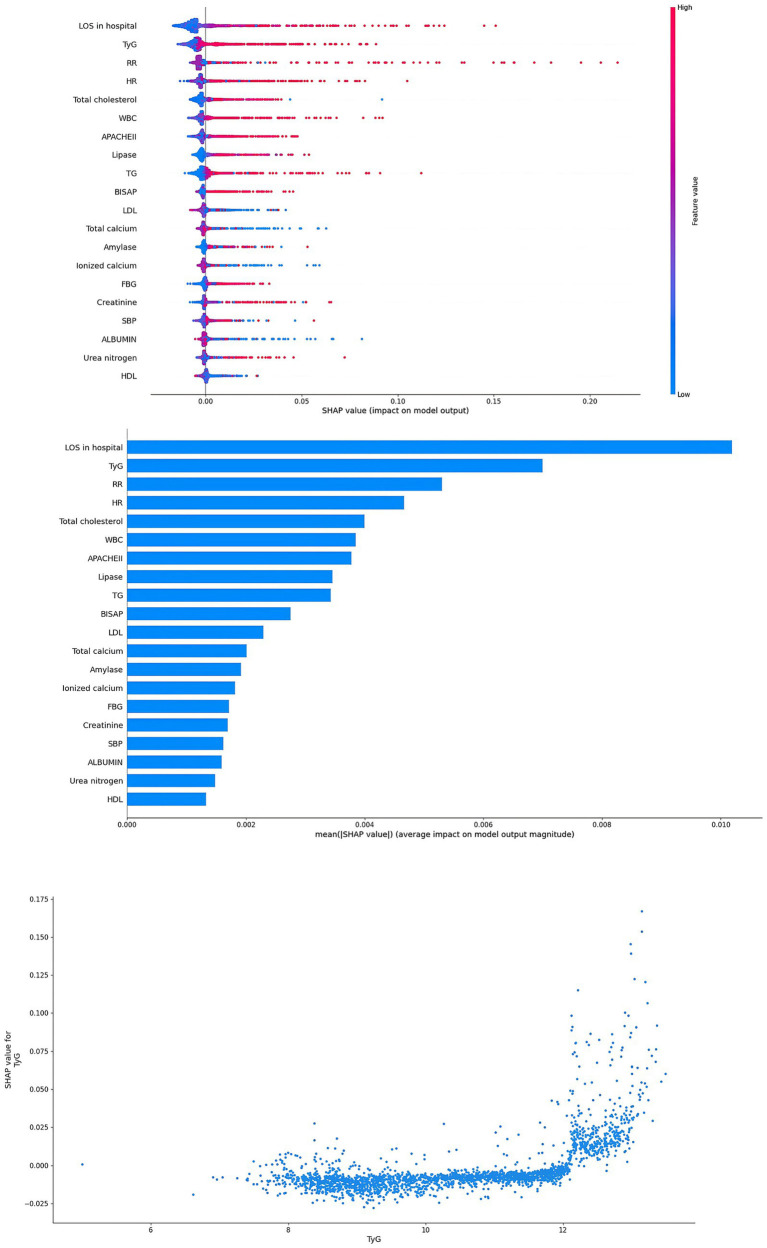
Interpretability for the RF model. TyG index, triglyceride glucose index; SBP, systolic blood pressure; HR, heart rate; RR, respiratory rate; TG, triglyceride; FBG, fasting blood glucose; WBC, white blood cells; HDL, high density lipoprotein; LDL, low density lipoprotein; LOS, length of stay; APACHE, acute physiology and chronic health evaluation; BISAP, bedside index of severity in acute pancreatitis.

**Figure 6:**
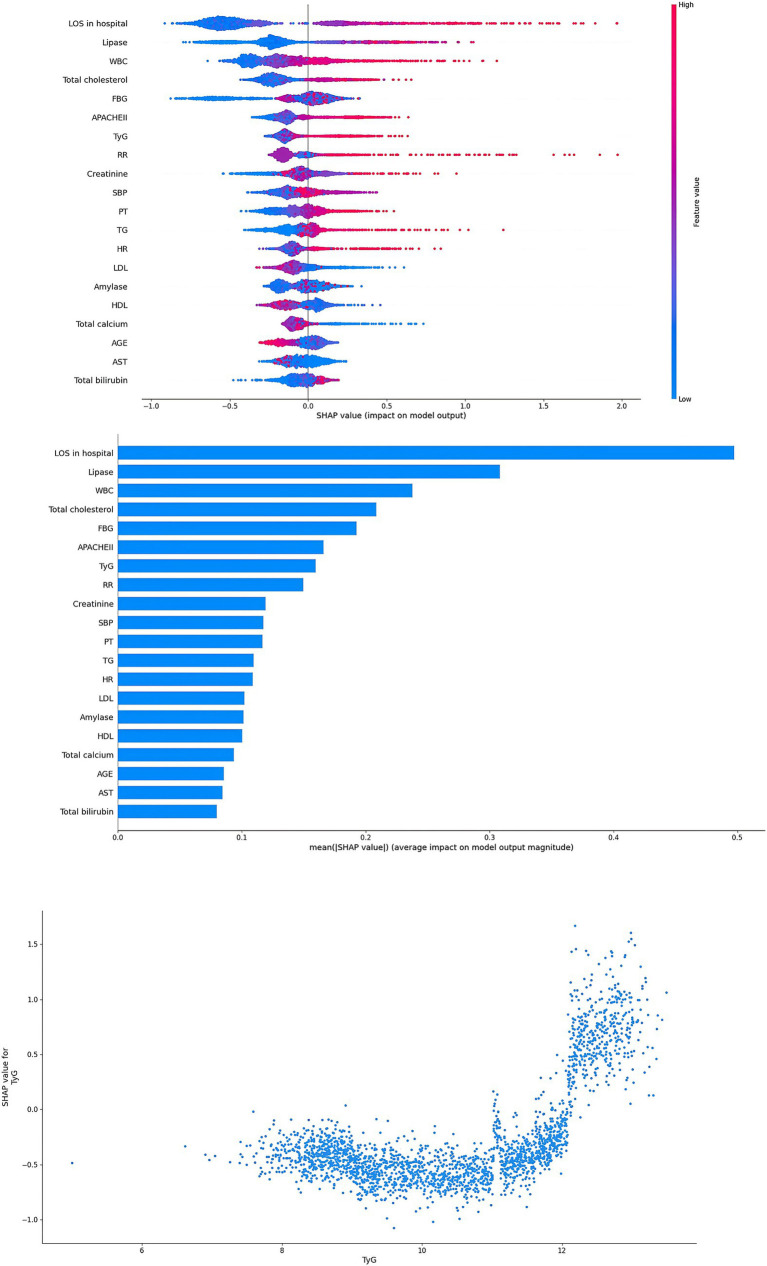
Interpretability for the XGBoost model. Abbreviations: TyG index, triglyceride glucose index; SBP, systolic blood pressure; HR, heart rate; RR, respiratory rate; TG, triglyceride; FBG, fasting blood glucose; WBC, white blood cells; PLT, platelet; PT, prothrombin time; AST, aspartate aminotransferase; LDL, low density lipoprotein; LOS, length of stay; APACHE, acute physiology and chronic health evaluation.

In this study, in order to verify the importance of the TyG, the SHAP method was applied to explain the ML models. In the RF model, the results showed that TyG was the second important factor (Figure 5). In addition, TyG was also one of the most important factors in the XGBoost model (Figure 6). In the SHAP method, each point represents a case. The higher the TyG is, the higher the corresponding SHAP value is, and the greater the contribution to the ARDS prediction.

## Discussion

Recent epidemiological analysis suggests that the incidence rate of hypertriglyceridemia acute pancreatitis (HTG-AP) is relatively high in China and is increasing year by year (13, 23). Compared with AP related to other etiologies, patients with HTG-AP have a more severe disease severity and a higher risk of life-threatening complications (24). This study included 2,382 patients with acute pancreatitis, of which HTG-AP accounted for 51.81%. In the ARDS group, the proportion of HTG-AP was higher, reaching 71.58%. This is consistent with previous literature. This may be due to the high levels of free fatty acids in HTG-AP patients, leading to fat embolism syndrome, which may result in pulmonary endothelial damage and microcirculation disorders (25).

The univariate analysis showed that a lower total calcium, ionized calcium, and HDL level, as well as a higher HR, RR, DBP, TyG, TG, FBG, WBC, PT, globulin, urea nitrogen, creatinine, amylase, lipase, and total cholesterol at admission were associated with a higher risk of developing ARDS in AP patients. Similar conclusions have been documented in prior studies (8, 26, 27).

AP patients with metabolic abnormalities such as hypertriglyceridemia, diabetes, and low HDL levels have a higher incidence of ARDS, suggesting that insulin resistance (IR) may play a role in the pathogenesis of ARDS. But the underlying mechanisms remain unknown.

IR can cause metabolic disorders, exacerbate oxidative stress in the body, enhance systemic inflammation, disrupt endothelial cell function, and stimulate the proliferation of vascular smooth muscle cells (28). These corresponding pathological changes may lead to ischemia–hypoxia-related damage to lung tissue, resulting in the occurrence and development of ARDS. Therefore, evaluating the IR of AP patients plays an important role in predicting the development of ARDS. The use of IR-related biomarkers, such as HOMA-IR index, cannot achieve accuracy because insulin measurement cannot distinguish between endogenous insulin and therapeutic exogenous insulin (29). Therefore, the TyG index has become a simple, reliable, and effective IR biomarker.

Research has indicated that there was a correlation between the TyG index and the severity of AP disease (13, 30). At the same time, the respiratory system is mainly affected, and ARDS is the most common form of organ failure. Therefore, our aim is to explore the relationship between the TyG index and ARDS in AP patients, with the aim of providing a new biomarker for identifying early ARDS in AP patients and promoting timely clinical management.

Interestingly, our research findings suggest a close and statistically significant association between the TyG index and ARDS in various models, including the crude model, adjusted model I, and model II. In addition, there is a non-linear relationship between them. When the TyG index is higher than 11.31, the incidence rate of ARDS increases significantly, indicating that there is a threshold effect. Previous research reports have shown a non-linear relationship between TyG index and arterial stiffness (31), major adverse cardiovascular events in patients with acute coronary syndrome (32), and the occurrence of acute respiratory failure in patients with AP (3). However, this study reports for the first time a non-linear association between the TyG index and ARDS risk in patients with AP.

In subgroup analysis, a higher incidence of ARDS was observed in men, individuals of lower age, and hypertriglyceridemia. This finding may be related to the higher prevalence of glycolipid metabolism disorders in younger individuals, which is frequently associated with unhealthy lifestyles such as alcohol consumption, physical inactivity, late-night eating, and sleep deprivation. Studies have shown that estrogen can ameliorate IR (33). 17*β*-Estradiol (E2) has been proven to be a key hormone signal for energy homeostasis, enhancing the adaptability of pancreatic islets to metabolic stress, improving the survival rate of pancreatic islet cells, and enhancing glucose-stimulated insulin biosynthesis and secretion effects. However, androgens may counteract this potential IR effect and benefit (3). This may explain why male AP patients have a higher risk of ARDS.

Our research indicates a significant correlation between the TyG index and the risk of ARDS in AP patients. Therefore, dynamically measuring the TyG index of AP patients can identify the risk of developing ARDS in AP patients at an early stage and intervene in the occurrence and development of ARDS through clinical treatment as early as possible. Of course, our research also has some limitations. First, as a single-center retrospective design study, it cannot establish a clear causal relationship with Ty. Despite conducting multivariate adjustments and subgroup analysis, residual confounding factors may still affect the results. In addition, this study only analyzed baseline TyG index levels and did not consider dynamic changes during hospitalization. Therefore, a larger multicenter prospective cohort design is needed to validate our findings and explore the predictive value of TyG index changes.

## Conclusion

TyG index was associated with hospital ARDS in AP patients. The XGBoost and Random Forest models based on the TyG index had the best performance for predicting ARDS in patients with AP. The SHAP method further confirmed that the TyG index serves as a significant predictor for the development of ARDS in patients with acute pancreatitis.

## Data Availability

The raw data supporting the conclusions of this article will be made available by the authors, without undue reservation.

## Footnotes

^1^ http://www.R-project.org

^2^ http://www.Empowerstats.com
