# Supplementary material for: Triglyceride–glucose index as a key predictor of ARDS in acute pancreatitis: SHAP analysis reveals its critical role in risk stratification
Source: Front Nutr. 2025 Nov 21;12:1662379. doi: 10.3389/fnut.2025.1662379 (PMC12679890; doi:10.3389/fnut.2025.1662379)
Supplement: Supplementary file 1 [file Image_1.pdf]

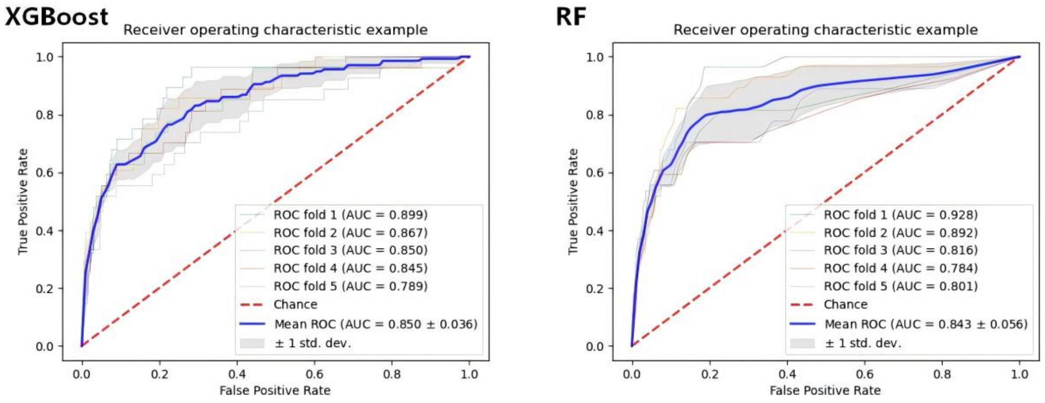

Figure S1. The model's performance after excluding TG and FBG

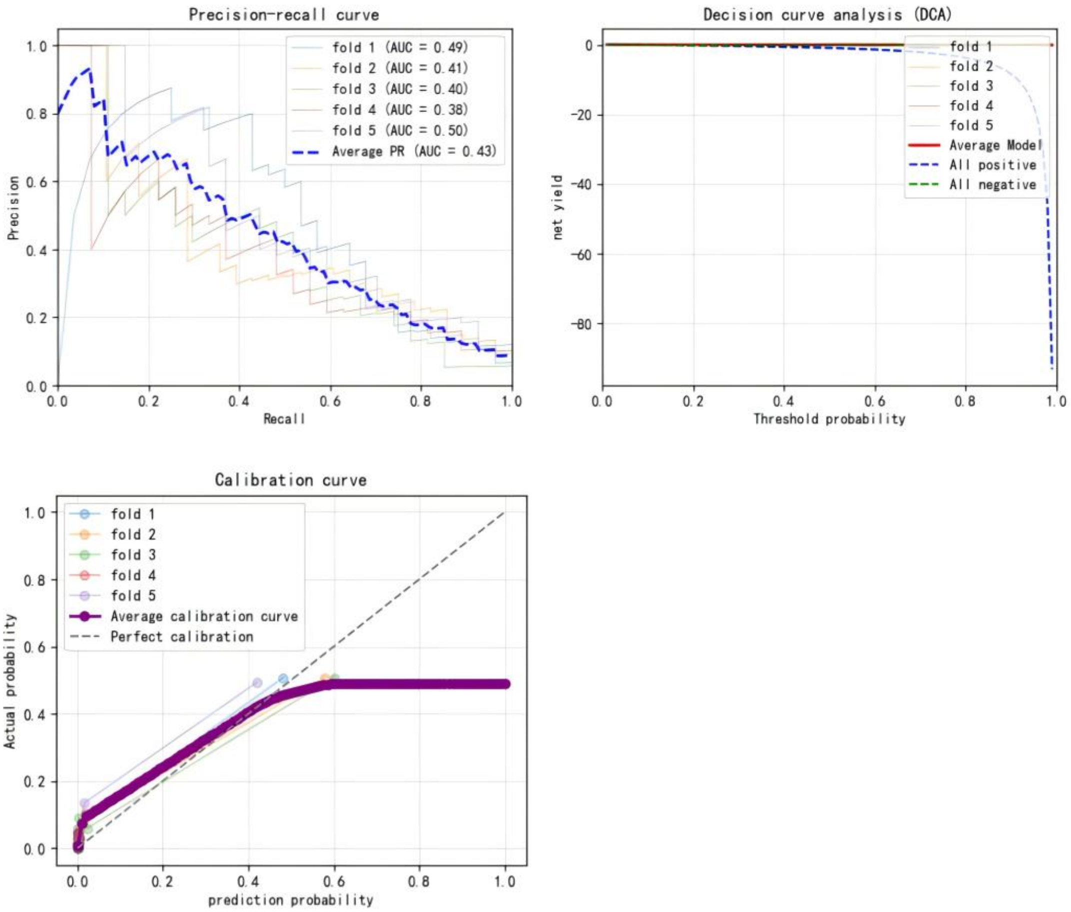

Figure S2. The Precision-Recall curve, Decision curve, and Calibration curve based on the XGBoost model

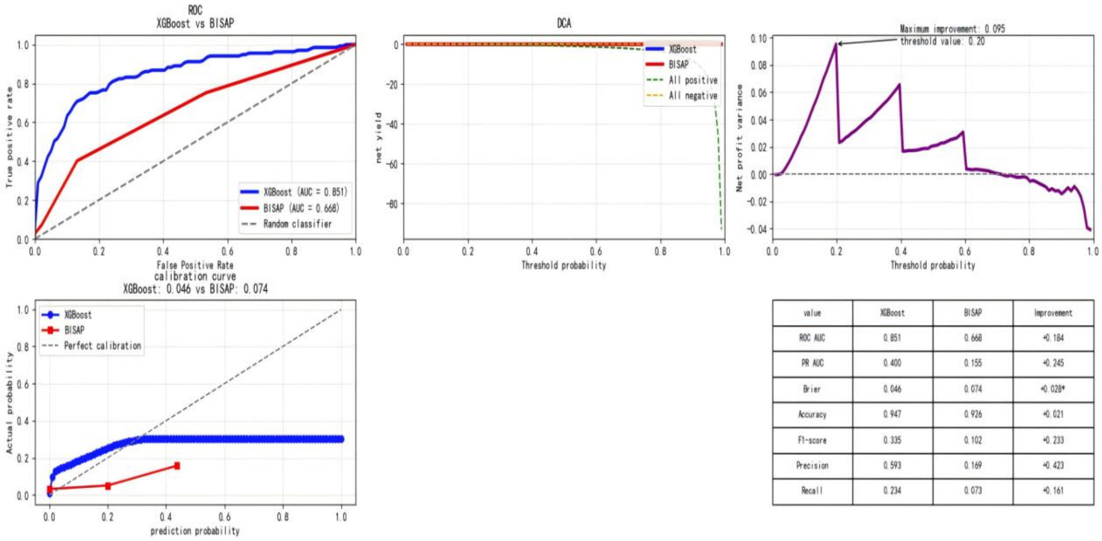

value	XGBoost	BISAP	Improvement
ROC AUC	0.851	0.668	+0.184
PR AUC	0.400	0.155	+0.245
Brier	0.046	0.074	+0.028*
Accuracy	0.947	0.926	+0.021
F1-score	0.335	0.102	+0.233
Precision	0.593	0.169	+0.423
Recall	0.234	0.073	+0.161

Figure S3. The XGBoost model outperformed the BISAP scoring system in predicting ARDS
